# The FDA-approved anti-amyloid-β monoclonal antibodies for the treatment of Alzheimer’s disease: a systematic review and meta-analysis of randomized controlled trials

**DOI:** 10.1186/s40001-023-01512-w

**Published:** 2023-11-28

**Authors:** Wenxue Wu, Yi Ji, Zilan Wang, Xiaoxiao Wu, Jiaxuan Li, Feng Gu, Zhouqing Chen, Zhong Wang

**Affiliations:** 1 Department of Neurosurgery & Brain and Nerve Research Laboratory, The First Affiliated Hospital of Soochow University, 188 Shizi Street, Suzhou, 215006 Jiangsu China; 2 Suzhou Medical College of Soochow University, Suzhou, 215002 Jiangsu China; 3 School of Medicine, Xiamen University, Xiamen, 361102 China

**Keywords:** Alzheimer’s disease, Aducanumab, Lecanemab, Anti-amyloid-β monoclonal antibodies

## Abstract

**Background:**

Alzheimer’s disease (AD) is a worldwide public health problem and is difficult to cure. Drugs aimed at slowing the progression of the disease have been developed, with the Food and Drug Administration (FDA) granting accelerated approval for aducanumab on June 21, 2021 and a new accelerated approval for lecanemab on January 22, 2023. We performed this systematic review and meta-analysis to assess the efficacy and safety of FDA-approved anti-amyloid-β (anti-Aβ) monoclonal antibodies (mabs) for the treatment of AD.

**Method:**

PubMed, Embase, and Cochrane Library were systematically searched to identify relevant studies published before May 2023. Efficacy outcomes included Aβ, neuroimaging, and biomarker outcomes. Safety outcomes included amyloid-related imaging abnormalities with edema or effusions (ARIA-E) and ARIA with cerebral microhemorrhages, cerebral macrohemorrhages, or superficial siderosis (ARIA-H). Review Manager 5.4 software was used to assess the data. The standard mean differences (SMDs) or odds ratio (OR) with 95% confidence interval (95% CI) were analyzed and calculated with a random effect model or a fixed effect model.

**Result:**

Overall, 4471 patients from 6 randomized controlled trials (RCTs), with 2190 patients in the treatment group and 2281 patients in the placebo group meeting the inclusion criteria. FDA-approved anti-Aβ mabs showed statistically significant improvements in clinical outcomes, including CDR-SB (P = 0.01), ADCS-ADL-MCI (P = 0.00003), ADCOMS (P < 0.00001), ADAS-Cog (P < 0.00001). Moreover, FDA-approved anti-Aβ mabs increased cerebrospinal fluid (CSF) Aβ1-42 (P = 0.002) and plasma Aβ42/40 ratios (P = 0.0008). They also decreased CSF P-Tau (P < 0.00001), CSF T-Tau (P < 0.00001), and plasma p-tau181 (P < 0.00001). FDA-approved anti-Aβ mabs perform neuroimaging changes in amyloid Positron Emission Tomography Standardized Uptake Value ratio (PET SUVr) (P < 0.00001). However, compared with placebo, FDA-approved anti-Aβ mabs had higher risk of ARIA-E (P < 0.00001) and ARIA-H (P < 0001).

**Conclusion:**

FDA-approved anti-Aβ mabs have a role in slowing disease progression in patients with AD, at the cost of an increased probability of side effects.

## Introduction

The latest data suggest the prevalence of Alzheimer’s disease (AD) will double in Europe and triple worldwide by 2050 [1]. It becomes a public health predicament in the world, and there is a significant impact on the direct cost of AD to the society.

Previously, the National Institute on Aging and Alzheimer’s Association classified the biomarkers of AD into A (amyloid), T (phosphorylated tau), and N (neurodegeneration): the ATN framework [2]. In other words, the main pathological change in AD is the accumulation of amyloid beta (Aβ) material in the brain, which can occur decades before the onset of clinical symptoms [1–3]. It may also induce downstream lesions, such as tau phosphorylation and aggregation, leading to neuronal death in AD [2, 4–8]. In addition, stages of AD can range from cognitively normal to mild cognitive impairment and dementia, which spans a period of years and emphasizes the continuity of the disease [1]. Therefore, it is important to diagnose and treat the disease early to slow down the disease process.

Currently, AD can be treated with non-pharmacologic therapy and pharmacologic therapy. Non-pharmacologic therapy consists of lifestyle changes, and multidomain interventions to prevent cognitive decline [6, 7, 9–11]. Pharmacotherapy is focused on disease-modifying treatments, including drugs targeting Aβ and Tau proteins, and other target classes such as proteostasis/ protein opathies, epigenetic regulators, synaptic plasticity and neuroprotection, inflammation and infection, metabolism and bioenergetics, vascular and growth factors are also of interest [1]. Among them, monoclonal antibodies (mabs) against tau proteins are aimed at binding to extracellular tau proteins, slowing or preventing their diffusion between cells and thus inhibiting tau protein aggregation and neurofibrillary tangle formation [12]. Phase II trials NCT02871921 and NCT03352557 were conducted to test the efficacy and safety of the semorinemab and gosuranemab. Whereas Aβ is the most common target in phase II and phase III drug development programs, only a few anti-amyloid-β (anti-Aβ) drugs have shown statistically significant cognitive benefits in AD clinical trials, despite a large body of evidence supporting the toxic effects of amyloid [13]. The anti-Aβ agents currently in clinical trials include: aducanumab, lecanemab, gantenerumab, donanemab, β-site Aβ precursor protein cleaving enzyme-1(BACE1), and BACE2, with NCT01760005, NCT03444870, NCT03443973, NCT05533411 all underway. Of all anti-Aβ approaches, passive immunotherapy using anti-Aβ mabs against Aβ has been best tolerated and given its mechanistic selectivity, it has been widely considered as the therapeutic candidate of choice [14]. These anti-Aβ mabs are also associated with downstream effects on tau pathology and neurodegeneration [15]. Among them, the FDA approved only two anti-Aβ mabs, aducanumab and lecanemab. Prior to this, only five drugs were approved by the FDA for clinical treatment, including acetylcholinesterase inhibitors and non-competitive *N*-methyl-d-aspartic acid (NMDA) receptor antagonists. However, these drugs are unable to alter AD progression, only for partial symptomatic relief [16]. Anti-Aβ drugs can slow the progression of the disease, probably because Aβ is more upstream in the overall pathological process, facilitating early treatment [15, 17].Although there have been several previous analyses of the safety and efficacy of anti-Aβ mabs for the treatment of AD, there have been no separate analyses of FDA-approved monoclonal antibodies. Critically, we included the recently reported lecanemab phase III results [18], which was the basis for the FDA’s accelerated approval. It is the second FDA approved anti-Aβ mabs for AD [19] and may have contributed to showing some statistically significant effects. Therefore, to provide evidence for clinicians, we pooled data from previous RCTs and conducted a meta-analysis to investigate the efficacy and safety of different FDA-approved anti-Aβ mabs for the treatment of AD.

## Method

### Search strategy

We followed the PRISMA guidelines for this systematic review and meta-analysis [20]. We searched Pubmed, Embase, and Cochrane Library until May 2023. The search strategy used included the following keywords: “AD”, “FDA”, “Alzheimer’s disease”, “lecanemab”, “BAN2401”, “aducanumab”, “aduhelm”, “BIIB037”, and “monoclonal antibody”.

### Selection criteria

Studies were included as follows: (1) Participant: patients with mild cognitive impairment (MCI) due to AD or mild AD dementia;(2) Intervention: patients treated with FDA-approved anti-Aβ mabs (lecanemab or aducanumab); (3) Comparison: patients treated with placebo; (4) Outcomes: Efficacy outcomes included clinical outcomes, neuroimaging and biomarker outcomes. Clinical outcomes included Clinical Dementia Rating Sum of Boxes (CDR-SB) which was the primary outcome and secondary outcomes such as Alzheimer’s Disease Cooperative Study-Activities of Daily Living Scale for Mild Cognitive Impairment (ADCS-ADL-MCI), Alzheimer’s Disease Composite Score (ADCOMS) and Alzheimer’s Disease Assessment Scale-Cognitive portion (ADAS-Cog). Amyloid Positron Emission Tomography Standardized Uptake Value ratio (PET SUVr) was the neuroimaging outcome. Biomarker outcomes included cerebrospinal fluid (CSF) levels of Aβ1-42, phosphorylated tau181 (p-tau), and total tau (t-tau), plasma Aβ42/40 ratio and plasma-tau181. Safety outcomes included amyloid-related imaging abnormalities (ARIA) with edema or effusions (ARIA-E) and ARIA with cerebral microhemorrhages, cerebral macrohemorrhages, or superficial siderosis (ARIA-H); (5) study design: double-blind placebo-controlled RCTs.

Studies were excluded as follows: (1) types of study were retrospective studies, cohort studies, reviews, meta-analysis, comments, and case reports; (2) not in English.

### Data extraction

All data were extracted separately by two independent authors, and disputes were resolved by a higher seniority author. We collected (1) baseline characteristics of the study, including author, year, and country; (2) patient characteristics, including number, types of drugs used for treatment; (3) efficacy of the drug, including clinical outcomes (CDR-SB, ADCS-ADL-MCI, ADCOMS, ADAS-Cog), neuroimaging data (amyloid PET SUVr), cerebrospinal fluid and plasma tests (CSF Aβ1-42, CSF p-tau, CSF t-tau, plasma Aβ42/40 ratio, plasma p-tau181); (4) safety of the drug, including ARIA-E and ARIA-H. The detailed data are listed in Table 1.

**Table 1:** Characteristics of the included studies and outcome events

Study	NCT	Countries	Centers	Publications	Number of participants (treatment vs. placebo)	Drug and dose	Male (%) (treatment vs. placebo)	Mean age ± SD (year) (treatment vs. placebo)	Study period	AD stage (MMSE score or global CDR score for inclusion)	MMSE, mean ± SD (treatment vs. Placebo)	CDR-SB score, mean ± SD (treatment vs. Placebo)	Outcome Events
van Dyck CH 2023	NCT03887455	USA	Multicenter	The New England Journal of Medicine	898 897	Lecanemab, 10 mg/kg	48.4 47.0	71.4 ± 7.9 71.0 ± 7.8	18 months	MCI due to AD or mild AD (Global CDR 0.5 or 1)	25.5 ± 2.2 25.6 ± 2.2	3.17 ± 1.34 3.22 ± 1.34	a, b, c, d, e, f, g, h, i, j, k, l
Swanson CJ 2022	NCT01767311	USA	Multicenter	Alzheimer's Research & Therapy	152 238	Lecanemab, 10 mg/kg	57.9 42.4	72.6 ± 8.8 71.1 ± 8.9	18 months	MCI due to AD or mild AD dementia (MMSE 22–28)	25.6 (2.4) 26.0 (2.3)	3.0 ± 1.4 2.9 ± 1.5	a, b, c, e, k, l
McDade E 2022	NCT01767311	USA	Multicenter	Alzheimer’s Research & Therapy	152 238	Lecanemab,10 mg/kg	57.9 42.4	72.6 ± 8.8 71.1 ± 8.9	18 months	MCI due to AD or mild AD (global CDR 0.5 or 1)	25.6 (2.4) 26.0 (2.3)	3.0 ± 1.4 2.9 ± 1.5	f, g
Budd Haeberlein, S EMERGE 2022	NCT02484547	USA	Multicenter	J Prev Alz Dis	547 548	Aducanumab, High dose (6 mg/kg (ApoEε4 +) or 10 mg/kg)	48 47	70.6 ± 7.5 70.8 ± 7.4	78week	MCI due to AD or mild AD dementia (MMSE 24–30 or global CDR 0.5)	26.3 ± 1.7 26.4 ± 1.8	2.51 ± 1.05 2.47 ± 1.00	a, b, d, e, g, h, i, j, k, l
Budd Haeberlein, S ENGAGE 2022	NCT02477800	USA	Multicenter	J Prev Alz Dis	555 545	Aducanumab, high dose (6 mg/kg (ApoEε4 +) or 10 mg/kg)	47 47	70.0 ± 7.7 69.8 ± 7.7	78week	MCI due to AD or mild AD dementia (MMSE 24 -30 or global CDR 0.5)	26.4 ± 1.8 26.4 ± 1.7	2.40 ± 1.01 2.40 ± 1.01	a, b, d, e, g, h, i, j, k, l
Sevigny, J 2016	NCT01677572	USA	Multicenter	Nature	32 40	Aducanumab,10 mg/kg	53 42	73.7 ± 8.3 72.8 ± 7.2	54week	prodromal to mild AD (MMSE 20–26 or global CDR 0.5 or 1)	24.8 ± 3.1 24.7 ± 3.6	3.14 ± 1.71 2.66 ± 1.50	a, e, k, l
Ferrero J 2016	NCT01397539	USA	Multicenter	Alzheimer’s & Dementia: TRCI	6 13	Aducanumab, 10 mg/kg	17 36	72.7 ± 4.5 66.9 ± 8.7	24week	mild-to-moderate AD (MMSE 14–26)	18.3 (4.9) 22.1 (2.4)		B

*AD* Alzheimer’s disease, *ARIA* amyloid-related imaging abnormalities, *MCI* Mild Cognitive Impairment, *MMSE* Mini-Mental State Examination, *SD* standard deviation

a: Clinical Dementia Rating–Sum of Boxes (CDR-SB); b: Alzheimer’s Disease Assessment Scale- Cognitive Subscale (ADAS-cog); c: Alzheimer’s Disease Composite Score (ADCOMS); d: Alzheimer’s Disease Cooperative Study–Activities of Daily Living Scale for Mild Cognitive Impairment (ADCS-MCI-ADL); e: Amyloid Burden on PET; f: plasma biomarker Aβ42/40 ratio; g: plasma biomarker p-tau181; h: CSF Aβ1-42; i: CSF T-Tau; j: CSF P-Tau; k: ARIA-H: ARIA with hemosiderin deposits; l: ARIA-E: ARIA with edema or effusions

### Outcome of interest

Efficacy outcomes included CDR-SB, ADCS-ADL-MCI, ADCOMS and ADAS-Cog for clinical assessment, amyloid PET SUVr, CSF Aβ1-42, CSF P-Tau, CSF T-Tau, plasma A β42/40 ratio and plasma p-tau181 for ancillary examinations (neuroimaging and biomarker outcomes). We used CDR-SB as the primary outcome, with a score range of 0–18, where a higher score represents a greater degree of impairment. Secondary endpoints include ADCS-ADL-MCI, ADCOMS, and ADAS-Cog, with lower scores on the ADCS-ADL-MCI and higher scores on ADCOMS and ADAS-Cog indicating more severe impairment. Whereas ADCS-ADL-MCI scores range from 0 to 53, ADCOMS scores range from 0 to 1.97 and ADAS-Cog scores range from 0 to 90.

Safety outcomes included ARIA-E and ARIA-H. ARIA-E refers to parenchymal edema and sulcal effusion. ARIA-H refers to deposits of hemosiderin (i.e., a blood degradation product), including parenchymal microhemorrhages, cerebral macrohemorrhages, and leptomeningeal superficial siderosis.

### Risk of bias

We assessed selection bias, performance bias, detection bias, attrition bias, reporting bias, and other potential biases using Review Manager 5.4 software (The Cochrane Collaboration, Oxford, UK). Two independent authors did this work, and the disagreement was resolved by a more senior author.

### Data analysis

The RCTs included in our meta-analysis contained two subgroups, which differed in drug names. To properly deal with variation between study subgroups, we followed the recommendation to treat subgroups as units of analysis, thus treating each subgroup as a separate study. All data were estimated using Review manager 5.4 to estimate standardized mean differences (SMD) or odds ratios (OR) and 95% confidence intervals (95%CI). Statistical heterogeneity was estimated using I^2^, with low heterogeneity being less than 50% and high heterogeneity being more than 50%. Random effects models were used for high heterogeneity, while fixed effects models were used for low heterogeneity. Subgroup analysis of individual drugs was performed. P-value < 0.05 indicates a statistically significant difference.

## Result

### Search results

**Fig. 1:**
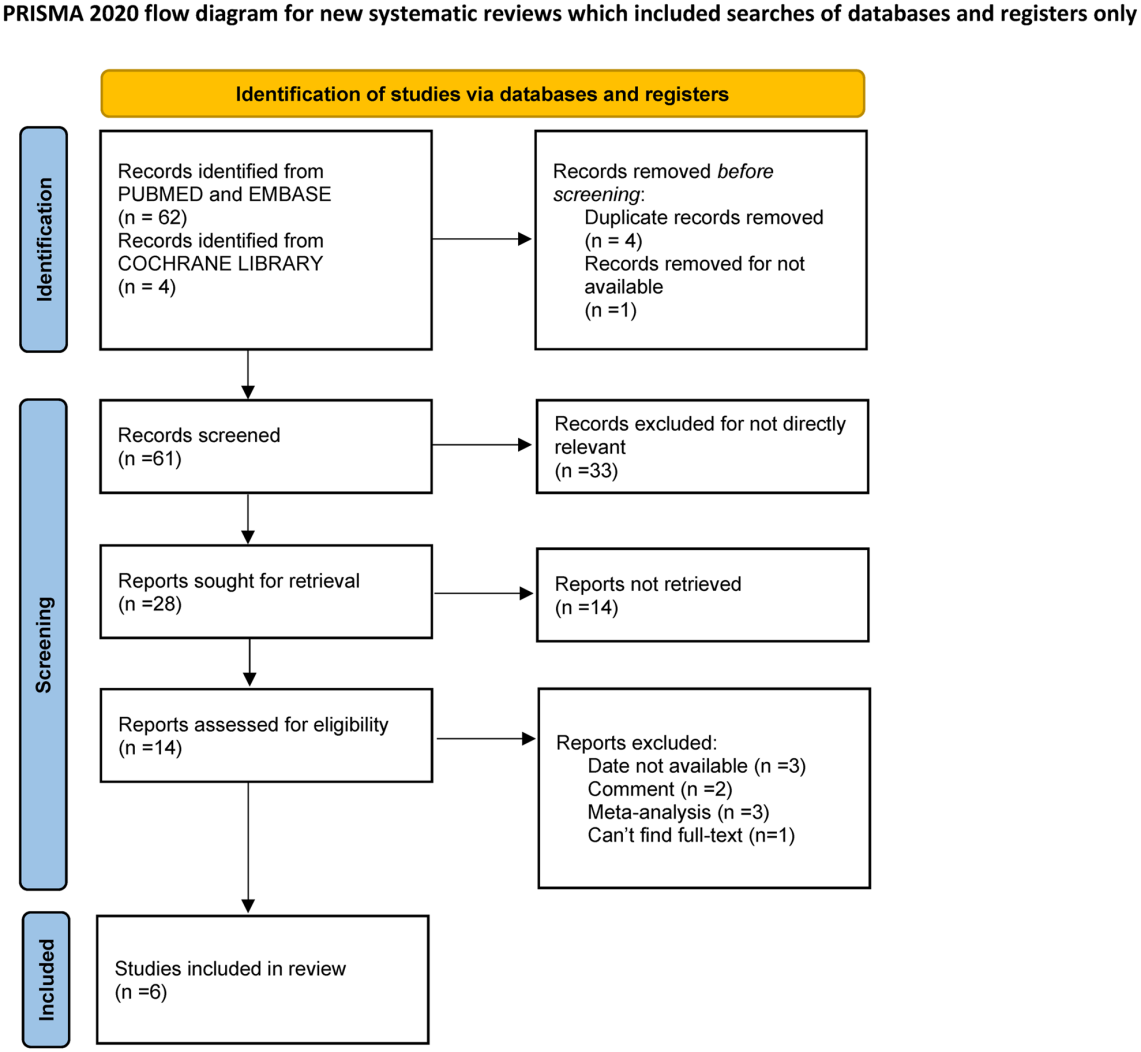
PRISMA flow diagram

We retrieved a total of 66 studies from online databases (Pubmed, Embase, Cochrane library). Four duplicates were removed. Based on the titles and abstracts, 33 irrelevant articles were excluded. For the remaining articles, after assessing the full text, studies with no data reported and types of studies such as retrospective studies and cohort studies were removed. Finally, we included 6 RCTs with a total of 4471 patients, including 2190 patients in the treatment group and 2281 patients in the placebo group. The detailed screening process is given in Fig. 1. Three studies tested lecanemab [18, 21] and three studies tested aducanumab [17, 22, 23]. The baseline characteristics of the patients are given in Table 1.

### Clinical outcomes

**Fig. 2:**
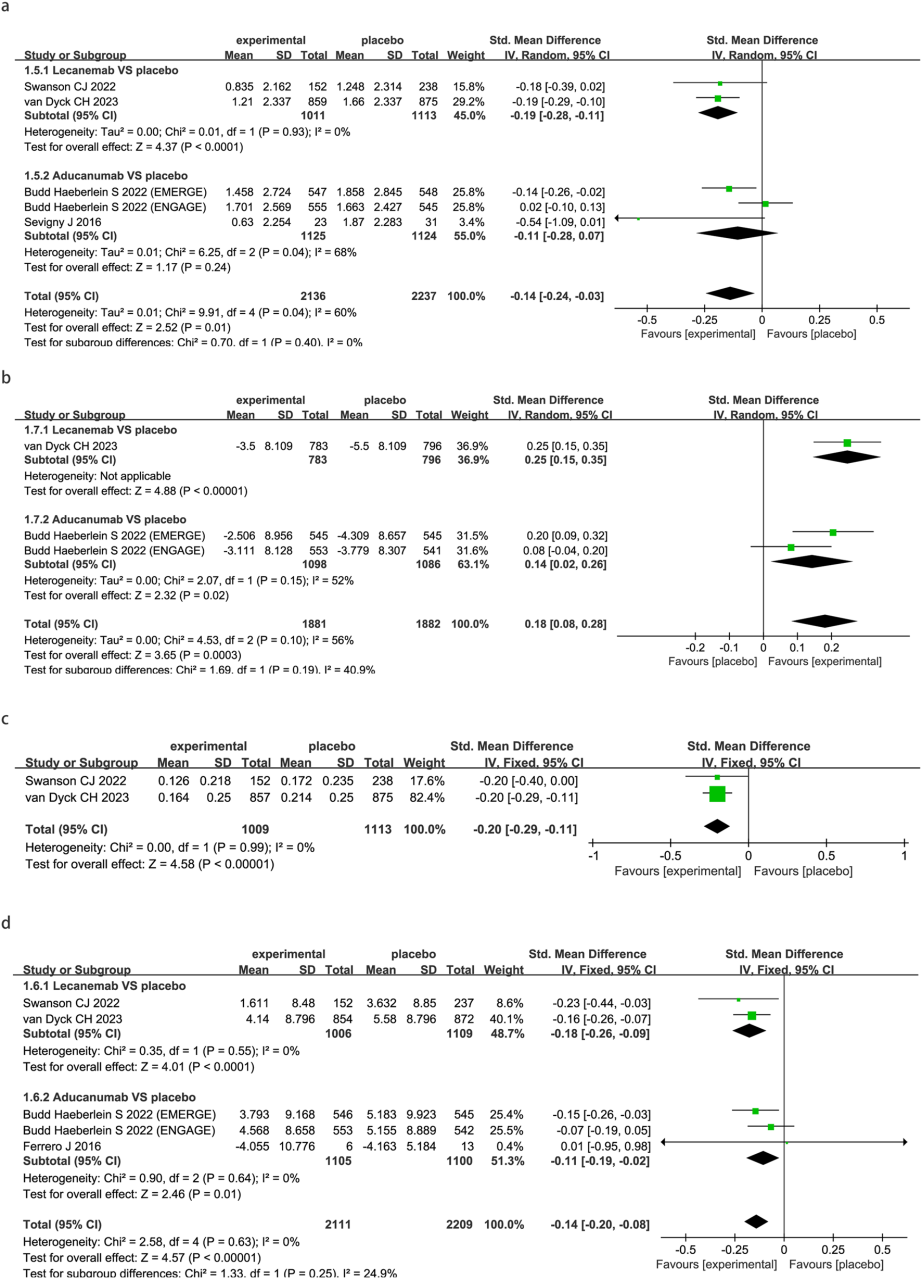
Meta-analysis of the clinical outcomes under anti-amyloid-β monoclonal antibodies in patients with AD. Forest plot showed the comparisons of mean changes between drugs and placebo on several tests: Changes in CDR-SB (**a**), Changes in ADCS-ADL-MCI (**b**), Changes in ADCOMS (**c**), and Changes in ADAS-Cog (**d**)

For the primary efficacy outcome CDR-SB, the FDA-approved anti-Aβ mabs statistically improved performance on the cognitive/functional measure CDR-SB (SMD − 0.14; 95% CI − 0.24 to − 0.03; P = 0.01, Fig. 2a). FDA-approved anti-Aβ mabs also had statistically improved ADCS-ADL-MCI (SMD 0.18; 95% CI 0.08 to 0.28; P = 0.0003, Fig. 2b) and ADCOMS (SMD − 0.20; 95% CI − 0.29 to − 0.11; P < 0.00001, Fig. 2c) as compared to the control group. Treatment with FDA-approved anti-Aβ mabs statistically improved performance on the cognitive measure ADAS-Cog score (SMD − 0.14; 95% CI − 0.20 to − 0.08; P < 0.00001, Fig. 2d) comparing with placebo.

Subgroup analysis by drug revealed that CDR-SB was statistically improved only by lecanemab (SMD − 0.19; 95% CI − 0.28 to − 0.11; P < 0.0001, Fig. 2a), whereas the efficacy of aducanumab was not significant (SMD − 0.11; 95% CI − 0.28 to 0.07; P = 0.24, Fig. 2a). Both lecanemab (SMD 0.25; 95% CI 0.15 to 0.35; P < 0.00001, Fig. 2b) and aducanumab (SMD 0.14; 95% CI 0.02 to 0.26; P = 0.02, Fig. 2b) statistically improved ADCS-ADL-MCI separately. Lecanemab showed statistical improvement for both ADCOMS (SMD − 0.20; 95% CI − 0.29 to − 0.11; P < 0.00001, Fig. 2c) and ADAS-Cog (SMD − 0.18; 95% CI − 0.26 to − 0.09; P < 0.0001, Fig. 2d). Aducanumab also showed statistical improvement for ADAS-Cog (SMD − 0.11; 95%CI − 0.19 to − 0.02; P = 0.01, Fig. 2d), while no data were available for ADCOMS.

### Neuroimaging and biomarker outcomes

**Fig. 3:**
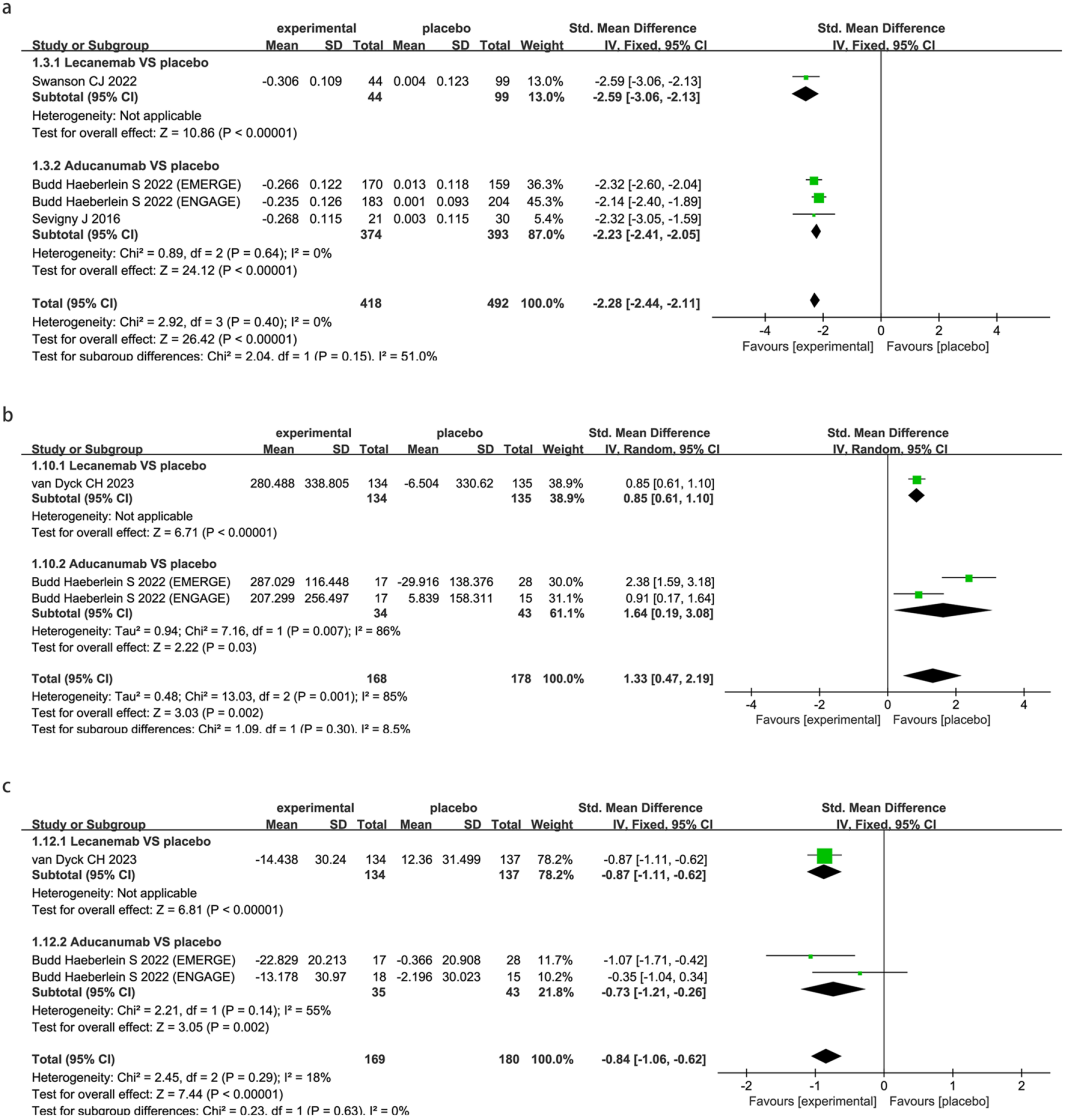

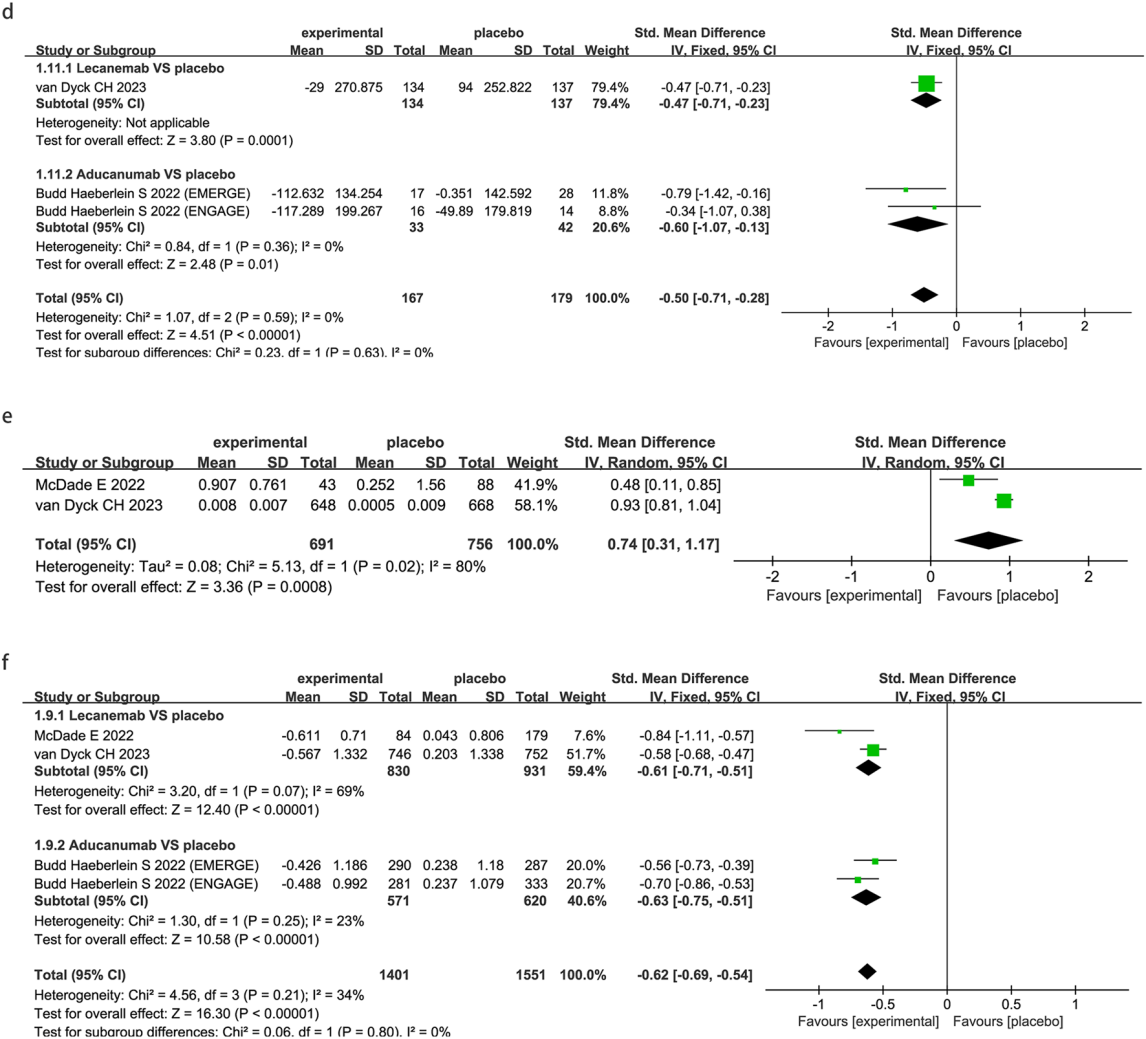
Meta-analysis of the neuroimaging and biomarkers outcomes under anti-amyloid-β monoclonal antibodies in patients with AD. Forest plot showed the comparisons of mean changes between drugs and placebo on neuroimaging and biomarkers outcomes:Changes in amyloid PET SUVr (**a**), Changes in CSF Aβ1-42 (**b**), Changes in CSF P-Tau (**c**), Changes in CSF T-Tau (**d**), Changes in plasma Aβ42/40 ratio (**e**), Changes in plasma p-tau181 (**f**)

Neuroimaging changes in AD patients (amyloid PET SUVr) are substantially reduced by FDA-approved anti-Aβ mabs (SMD − 2.28; 95% CI − 2.44 to − 2.11; P < 0.00001, Fig. 3a), subgroup analysis indicated both lecanemab (SMD − 2.59; 95% CI − 3.06 to − 2.13; P < 0.00001, Fig. 3a) and aducanumab (SMD − 2.23; 95% CI − 2.41 to − 2.05; P < 0.00001, Fig. 3a) significantly reduced amyloid PET SUVr.

The FDA-approved anti-Aβ mabs statistically increased Aβ1-42 (SMD 1.33; 95% CI 0.47 to 2.19; P = 0.002, Fig. 3b) while statistically decreased P-Tau (SMD − 0.84; 95% CI − 1.06 to − 0.62; P < 0.00001, Fig. 3c) and T-Tau (SMD − 0.50; 95% CI − 0.71 to − 0.28; P < 0.00001, Fig. 3d) in CSF. Subgroup analysis by drug showed that Aβ1-42 was statistically increased by lecanemab (SMD 0.85; 95% CI 0.61 to 1.10; P < 0.00001, Fig. 3b) and aducanumab (SMD 1.64; 95% CI 0.19 to 3.08; P = 0.03, Fig. 3b) separately. P-Tau (SMD − 0.87; 95% CI − 1.11 to − 0.62; P < 0.00001, Fig. 3c) and T-Tau (SMD − 0.47; 95% CI − 0.71 to − 0.23; P = 0.0001, Fig. 3d) were statistically decreased after treated with lecanemab. Also, P-Tau (SMD − 0.73; 95% CI − 1.21 to − 0.26; P = 0.002, Fig. 3c) and T-Tau (SMD − 0.60; 95% CI − 1.07 to − 0.13; P = 0.01, Fig. 3d) were significantly decreased after treatment with aducanumab.

For substances of interest in plasma, lecanemab statistically increased Aβ42/40 ratio (SMD 0.74; 95% CI 0.31 to 1.17; P = 0.0008, Fig. 3e) while aducanumab lacked experimental data to support the effect for Aβ42/40 ratio. The FDA-approved anti-Aβ mabs showed significant decrease in p-tau181 (SMD − 0.62; 95% CI − 0.69 to − 0.54; P < 0.00001, Fig. 3f). Subgroup analysis by drug showed that lecanemab (SMD − 0.61; 95% CI − 0.71 to − 0.51; P < 0.00001, Fig. 3f) and aducanumab (SMD − 0.63; 95% CI − 0.75 to − 0.51; P < 0.00001, Fig. 3f) separately reduced p-tau181.

### Safety outcomes

**Fig. 4:**
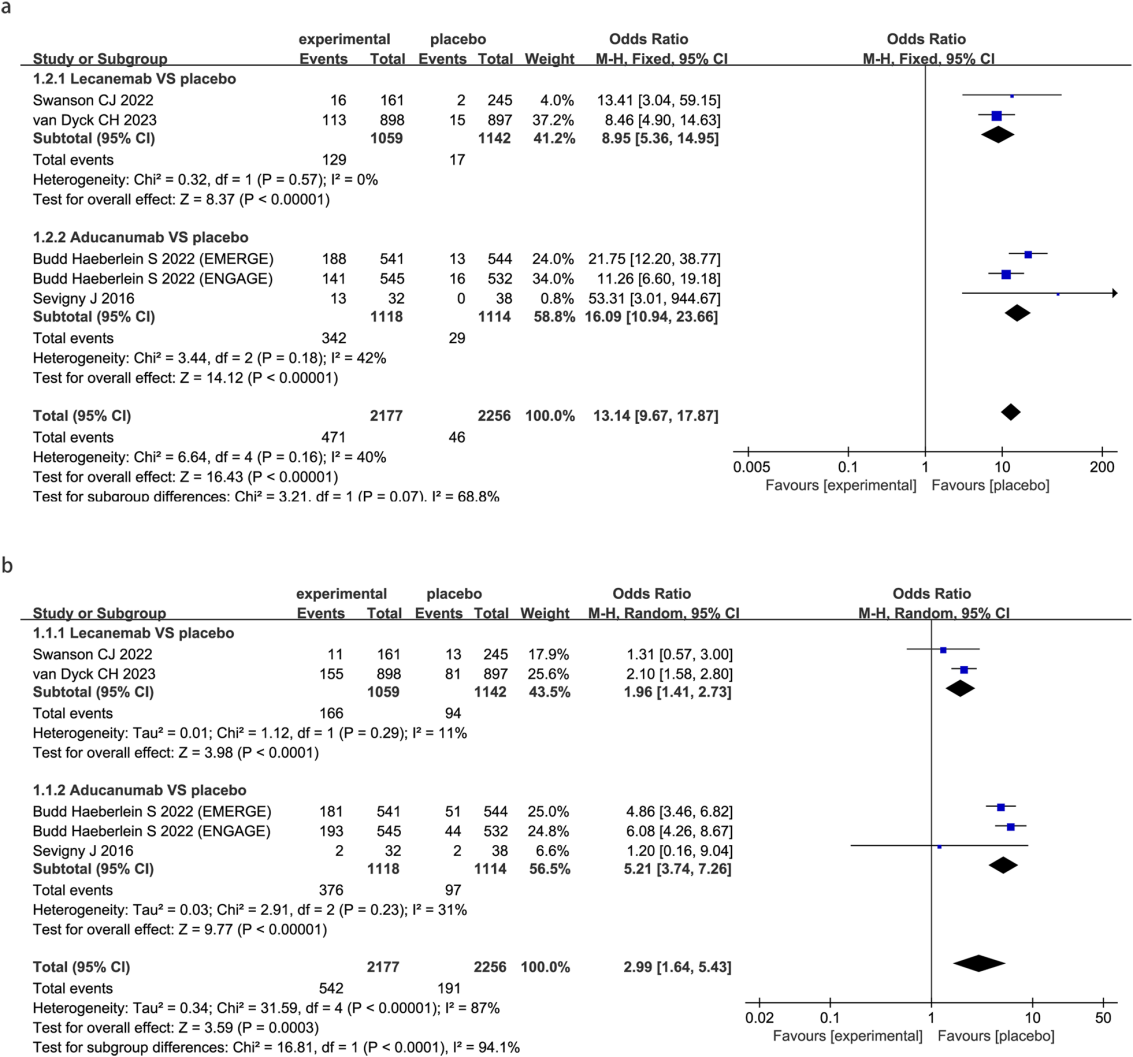
Meta-analysis of the safety outcomes under anti-amyloid-β monoclonal antibodies in patients with AD. Forest plot of comparisons between drugs and placebo on ARIA-E (**a**) and ARIA-H (**b**)

To note, compared with placebo, FDA-approved anti-Aβ mabs substantially increased the risk of ARIA-E (OR 13.14; 95% CI 9.67 to 17.87; P < 0.00001, Fig. 4a) and ARIA-H (OR 2.99; 95% CI 1.64 to 5.43; P < 0001, Fig. 4b).

Subgroup analysis by drug showed that lecanemab significantly increased the risk for ARIA-E (OR 8.95; 95% CI 5.36 to 14.95; P < 0.00001, Fig. 4a) and ARIA-H (OR 1.96; 95% CI 1.41 to 2.73; P < 0.0001, Fig. 4b). aducanumab significantly increased the risk for ARIA-E (OR 16.09; 95% CI 10.94 to 23.66; P < 0.00001, Fig. 4a) and ARIA-H (OR 5.21; 95% CI 3.74 to 7.26; P < 0.00001, Fig. 4b).

### Risk of bias

**Fig. 5:**
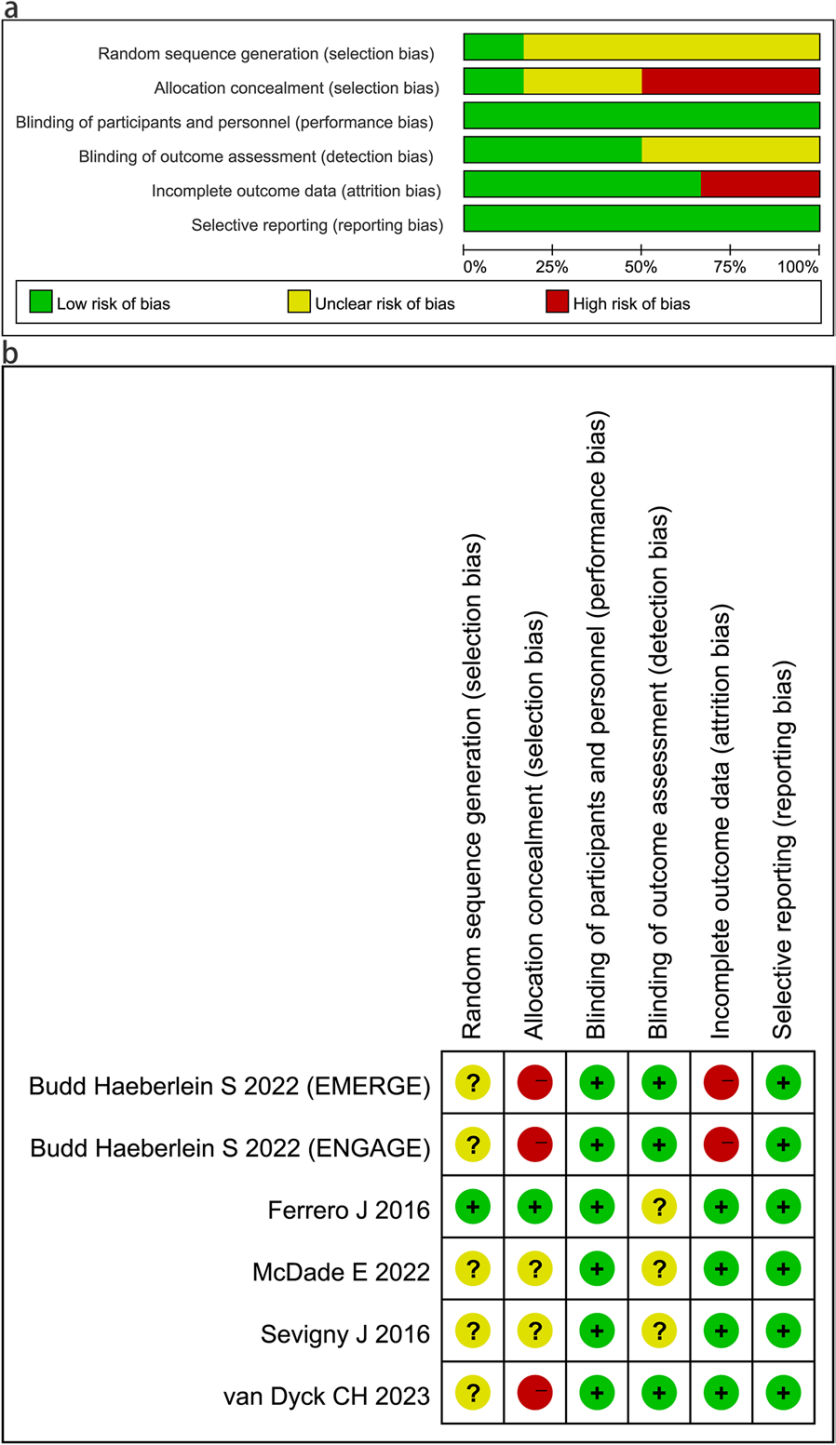
Summary of bias risk assessment results and quality of the included RCTs

Details of the risk of bias for each of the included RCTs are in Fig. 5. For random sequence generation, the risk of bias for the 5 studies was unclear. For allocation concealment, the risk of bias for the 2 studies was unclear and 3 studies were at high risk of bias. For blinding of participants and personnel and selective reporting, the risk of bias was low for all 6 studies. For the blinding of outcome assessment, the risk of bias was unclear for 3 trials. For incomplete outcome data, the risk of bias was high for 2 studies.

## Discussion

FDA-approved lecanemab and aducanumab are anti-Aβ mabs that can slow the disease process of AD [18], targeting the pathophysiological mechanisms of AD. This is the first meta-analysis of the efficacy and safety of only these two FDA-approved drugs. We found statistically significant improvements in clinical outcomes (CDR-SB, ADCS-ADL-MCI, ADCOMS, ADAS-Cog), neuroimaging (amyloid PET SUVr), and biomarkers (CSF Aβ1-42, CSF P-Tau, CSF T-Tau, plasma A β42/40 ratio, plasma p-tau181) with lecanemab. There was no statistically significant difference in CDR-SB for aducanumab compared with placebo. Conversely, aducanumab contributed to the ADCS-ADL-MCI, ADAS-Cog, neuroimaging, and biomarkers outcomes improvements, except for the absence of accessible data for ADCOMS and plasma Aβ42/40 ratio. Both drugs had elevated adverse effects compared to placebo, which means they were more aggressive.

Prior to 2003, the FDA approved only five drugs for the treatment of AD: tacrine, donepezil, rivastigmine, galantamine and memantine. The first four are acetylcholinesterase (AChE) inhibitors, and memantine is an *N*-methyl-d-aspartic acid (NMDA) receptor-holding agent. All of these drugs only relieve symptoms and do not slow disease progression. In June 2021, the FDA announced accelerated approval of aducanumab, the first drug approved to slow the progression of AD, and another new FDA approval for AD in nearly 20 years. The first drug used to slow the progression of AD [18, 24]. Aducanumab is a human mab that selectively targets aggregated forms of Aβ, including soluble oligomers and insoluble fibrils [17]. Despite the FDA approval, the effectiveness of aducanumab remains controversial. A phase III clinical trial by Budd et al. [22] was used to test the efficacy of aducanumab. These included two large trials, ENGAGE with 1653 patients and EMERGE with 1643 patients, but trials were terminated early due to the outcome of a futility analysis. One reason for discontinuing the trials was that the primary endpoint (CDR-SB) in ENGAGE was not met. However, no evidence has shown that the early termination of the studies affected the integrity or validity of the results or conclusions from either study. The robustness of the study results was demonstrated by sensitivity and supplementary analyses [22]. In fact, the final data from these two studies showed a greater magnitude of treatment effect compared to the invalid interim data. It is noteworthy that aducanumab caused a large reduction in brain Aβ at the cost of a higher ARIA compared to lecanemab. The study by Jeong et al. also reported a higher incidence of adverse events with aducanumab compared to other mabs. The reason for this may be attributed to different biological mechanisms by which different types of mabs target Aβ, as well as their different selectivity for antibody solubility [25]. Aducanumab partially targets oligomers, while primarily clearing insoluble amyloid plaque, which is associated with vasogenic brain edema, raising the risk of adverse effects.

Subsequent to the FDA’s recent approval of lecanemab in January 2023, supported by a clinical research published in February 2023 [19], we performed this meta-analysis and found for the first time that lecanemab may have better efficacy than aducanumab. Possible reason for the great extent of ameliorative effect may be that lecanemab is a humanized IgG1 anti-Aβ mabs and can selectively bind to large, soluble Aβ protofibrils that are the most neurotoxic and contribute to the pathogenesis of AD [26]. The trial to speed up lecanemab approval was a multicenter, double-blind, phase III trial, with the primary endpoint of CDR-SB at 18 months. At 18 months, the primary regression indicator CDR-SB changed less from baseline to the end of follow-up in the lecanemab group compared to the placebo group, while the remaining indicators (amyloid, tau protein, neurodegenerative lesions) decreased more [18]. Compared to aducanumab, lecanemab had a lower risk of side effect, possibly reason was that it selectively targets the soluble conformation of Aβ (i.e., does not bind to plaque) [13, 27]. According to our study, all clinical outcomes were mildly improved. Similar to our findings, a previous review concluded that mabs statistically improved cognition with small effect sizes and vigorously reduced brain amyloid burden, but increased the risk of ARIA [8]. However, this review lacked the data analysis of lecanemab.

As for neuroimaging, PET SUVr is the only imaging data available for the assessment of Aβ deposition by PET. Previous studies have shown that assessing enrichment of Aβ plaque load is particularly relevant in assessing the feasibility of clinical trials in enriched amyloid-positive patients with AD, where separate clinical criteria appear to lead to serious misclassification [28]. This is in line with the current trend of AD diagnosis and treatment. In the context of the imaging boom, PET-CT can help increase the possibility of early diagnosis of AD and help patients receive treatment before symptoms appear for a better quality of life. In addition, CSF (Aβ1-42, T-Tau, P-Tau) and plasma (p-tau181, Aβ42/40 ratio) from selected patients were collected and analyzed together, and it was found that changes in biomarkers may be sequential in AD patients [22]. Previous studies have shown that an increase in Aβ plaques occurs first, followed by an increase in soluble p-tau levels, which in turn may lead to the accumulation of neurofibrillary tangles (NFTs) and subsequent cognitive decline [29]. Therefore, targeting the upstream of AD pathogenesis for the earlier efficacy to slow down the disease process.

We also have some limitations. Most notably, the number of RCTs we included was small and sample size varied differently. In addition, we only analyzed data from the experimental group at a single dose (10 mg/kg) and failed to take into account the effects of different doses on outcomes, which may reduce the credibility of the results. We chose this single dose (10 mg/kg) because it was the only dose that all of the RCTs included, and it has been identified as an appropriate dose [17]. Moreover, in the most recent and largest RCT, only a biweekly 10 mg/kg dose of lecanemab was used to treat early AD [18]. We performed subgroup analyses of the different outcome indicators according to the therapeutic agents of the included patients. However, subgroup analyses were not performed according to different populations (e.g., women, APOE e4 homozygous carriers), in which the effects may be different than in the whole sample (see, for example, the supplementary material of the van Dyck et al. lecanemab phase III RCT. Another limitation is that the effect of aducanumab on structural MRI (greater ventricular enlargement compared with placebo) was not considered in this review. Greater atrophy induced by these drugs is a potential concern.

Although the FDA approved two drugs to slow the disease process, the safety of these two drugs is yet to be considered and more clinical trials are expected to prove this.

## Conclusion

This meta-analysis showed that FDA-approved anti-Aβ mabs statistically improved clinical outcomes and neuroimaging, and statistically changed the levels of biomarkers, suggesting a role for both drugs in slowing disease progression in AD patients, but at the cost of an increased probability of side effects. From this meta-analysis, we found for the first time that lecanemab may have better efficacy than aducanumab. These results offer new hope for the development of anti-Aβ mabs. We also hope that these results will provide a reference for the discovery of targeting the pathological mechanisms of AD, with the aim of developing more effective drugs that can modify the disease process of AD.

## Data Availability

The datasets used and/or analyzed during the current study are available from the corresponding author on reasonable request.

## Abbreviations

AD: Alzheimer’s disease
RCTs: Randomized controlled trials
FDA: Food and Drug Administration
Aβ: Amyloid beta
anti-Aβ: Anti-amyloid-β
mabs: Monoclonal antibodies
BACE1: β-Site Aβ precursor protein cleaving enzyme-1
BACE2: β-Site Aβ precursor protein cleaving enzyme-2
NMDA: Non-competitive *N*-methyl-d-aspartic acid
MMSE: Mini-Mental State Examination
CDR-SB: Clinical Dementia Rating-Sum of Boxes
ADCS-ADL-MCI: Alzheimer’s Disease Cooperative Study-Activities of Daily Living Scale for Mild Cognitive Impairment
ADCOMS: Alzheimer’s Disease Composite Score
ADAS-Cog: Alzheimer’s Disease Assessment Scale-Cognitive Subscale
PET: Positron Emission Tomography
SUVr: Standardized Uptake Value ratio
CT: Computed Tomography
p-tau: Phosphorylated tau
t-tau: Total tau
ARIA-E: Amyloid-related imaging abnormalities with edema or effusions
ARIA-H: ARIA with cerebral microhemorrhages, cerebral macrohemorrhages, or superficial siderosis
CSF: Cerebrospinal fluid
NFTs: Neurofibrillary tangles
SMDs: Standard mean differences
OR: Odds ratio
95% CI: 95% Confidence interval
